# Serum prohepcidin levels are potential prognostic markers in patients with multiple myeloma

**DOI:** 10.3892/etm.2012.663

**Published:** 2012-08-18

**Authors:** KOUICHI HARAGUCHI, HIROFUMI UTO, NOBUHITO OHNOU, MASAHITO TOKUNAGA, MAYUMI TOKUNAGA, ATAE UTSUNOMIYA, SHUICHI HANADA, HIROHITO TSUBOUCHI

**Affiliations:** 1 Digestive and Lifestyle Diseases, Department of Human and Environmental Sciences, Kagoshima University Graduate School of Medical and Dental Sciences, Kagoshima 890-8544;; 2 Department of Hematology, National Hospital Organization Kagoshima Medical Center, Kagoshima 892-0853;; 3 Department of Hematology, Ikeda Hospital, Kanoya 893-0024;; 4 Department of Hematology, Imamura Bun-in Hospital, Kagoshima 890-0064, Japan

**Keywords:** hepcidin, prohepcidin, multiple myeloma, prognostic factor

## Abstract

Prohepcidin is the prohormone of hepcidin. Anemia is one of the main clinical features in patients with multiple myeloma (MM) and hepcidin may be associated with iron homeostasis in these patients. However, the clinical significance of prohepcidin is not fully understood. In this retrospective study, we measured serum prohepcidin levels using an immunoassay technique to study its clinical significance in 39 MM patients. Serum prohepcidin levels in patients with MM were weakly correlated with alkaline phosphatase (ALP) levels (r=0.32, P=0.048), calculated by Spearman’s rank correlation, but not with other clinical data, including hemoglobin, serum iron or ferritin. In addition, patients with severe renal insufficiency [creatinine clearance (CCr) <50 ml/min] had significantly higher prohepcidin levels compared with patients with mild or no renal insufficiency (CCr ≥50 ml/min, P=0.047). In contrast, low serum prohepcidin levels less than 110 ng/ml were an independent predictor of poor overall survival [hazard ratio (HR), 5.29; 95% confidence interval (CI), 1.65–17.03] in addition to serum creatinine levels of at least 2 mg/dl (HR, 5.32; CI, 1.10–25.64), serum calcium (HR, 3.53; CI, 1.01–12.33) and ECOG performance status grade 4 (HR, 4.15; CI, 1.32–13.09) in the multivariate analysis using Cox proportional hazards model. In the subset of 31 MM patients with CCr ≥50 ml/min, low serum prohepcidin (HR, 5.65; CI, 1.60–19.95) was an indicator of poor prognosis in multivariate analysis. These results indicate that serum prohepcidin levels may be associated with ALP and renal function but not iron homeostasis, in MM patients. In addition, lower serum prohepcidin levels are potential independent indicators of poor overall survival in MM patients regardless of renal function.

## Introduction

Multiple myeloma (MM) is a B cell lymphoproliferative disorder characterized by monoclonal proliferation of plasma cells with overproduction of a specific monoclonal antibody (1). Symptomatic MM patients usually present with at least one of the following complications: anemia, hypercalcemia, renal insufficiency or bone lesions. Anemia may be the most common single clinical feature of MM, being present in appoximately 73% of patients at diagnosis (2). The anemia in patients with MM is usually normocytic and normochromic, with normal to high serum iron levels and high serum ferritin levels (3).

The main mediator of anemia in patients with chronic disease is considered to be the regulatory hormone, hepcidin. Hepcidin, an antimicrobial peptide (4), is produced predominantly by hepatocytes (5). Cleavage of the signal peptide from the hepcidin precursor protein yields the 60 amino acid prohepcidin molecule. The biologically active 25 amino acid hepcidin molecule is generated by the removal of the proregion by the prohormone convertase furin and other members of the furin family (6). Mature hepcidin regulates iron absorption and distribution by binding to ferroportin, which is the exporter of iron in enterocytes and macrophages (7). Hepcidin assays based on mass spectroscopic techniques (8,9) or immunoassays (10–12) have been developed, and ELISA is available to measure serum levels of prohepcidin (13). However, there is no consensus on which are the best assays for measuring hepcidin levels and it has not been confirmed whether serum prohepcidin accurately reflects active hepcidin levels.

Hepcidin synthesis is markedly increased by infection and inflammation (14–16), and these effects are mediated by inflammatory cytokines, including interleukin (IL)-1 and IL-6 (16,17). IL-6 levels are increased in MM (18). Hepcidin is also reported to be upregulated in MM patients by IL-6-dependent and -independent mechanisms, and may play a role in the development of anemia (19). However, the associations between serum prohepcidin and inflammation or iron metabolism are controversial (14,20–22), and the clinical significance of prohepcidin levels in MM patients is not fully understood. In this study, we analyzed serum prohepcidin levels to determine its significance in patients with MM.

## Materials and methods

### Patients

A total of 39 patients with clinical Durie-Salmon stage II or III MM (23) (mean age, 66.7±10.9 years; 22 males, 17 females) were enrolled in this retrospective study (Table I). All patients were newly diagnosed prior to receiving chemotherapy between April 2000 and May 2008. The clinical stage of patients according to the Durie-Salmon Staging System and the International Staging System (ISS) (24) are shown in Table I. The prognostic indicators of MM (24), including age, platelet count, bone marrow plasma cell percentage, serum levels of hemoglobin, lactate dehydrogenase (LDH), creatinine, calcium, albumin, serum β2-microglobulin (sβ2M) and Eastern Cooperative Oncology Group (ECOG) performance status at diagnosis were examined. Creatinine clearance (CCr) and estimated glomerular filtration rate (eGFR) were also examined. Eighteen healthy subjects were enrolled as normal controls in the present study (mean age, 51.9±10.4 years; 14 males, 4 females; mean CCr, 82.7±17.5 ml/min; range of CCr, 61.0–129.0). Prognosis was assessed by the overall survival. The endpoint was considered as mortality, the last available follow-up date or September 30, 2008. This study was approved by the ethics committees of Kagoshima University Medical and Dental Hospital, and National Hospital Organization Kagoshima Medical Center (Kagoshima, Japan).

### Serum prohepcidin, IL-1β, IL-6 and tumor necrosis factor (TNF)-α measurement

The serum prohepcidin concentrations were determined by ELISA using a commercially available kit (DRG Instruments, Marburg, Germany) according to the manufacturer’s instructions. The lower detection limit of this assay was 3.95 ng/ml.

The levels of IL-1β, IL-6 and TNF-α in available serum samples were measured with ELISA kits (R&D Systems, Minneapolis, MN, USA), with lower limits of detection at 3.9 pg/ml, 3.13 pg/ml and 15.6 pg/ml, respectively.

### Statistical analysis

The values are expressed as means ± SD. The differences between groups were analyzed with the Mann-Whitney U test and the correlations between continuous variables were calculated by Spearman’s rank correlation. The Kaplan-Meier method was used to estimate survival and the log-rank test was used to compare the groups for differences in prognoses. Survival durations were measured between the date when chemotherapy was initiated and the endpoint. The stepwise Cox proportional hazards model was used in the multivariate analyses. P<0.05 was considered to indicate a statistically significant result. Statistical analysis was performed using SPSS, version 12.0 (SPSS Inc., Chicago, IL, USA).

## Results

### Serum prohepcidin levels in MM patients

**Figure 1:**
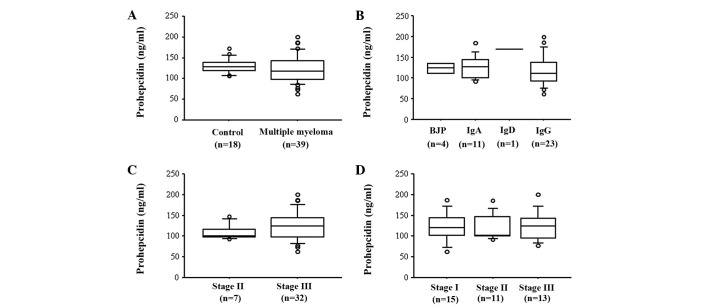
Serum prohepcidin levels in patients with multiple myeloma (MM). (A) There was no significant difference in serum prohepcidin levels between patients with MM and healthy controls (P=0.11). Serum prohepcidin levels in patients with MM were similar among subgroups according to (B) immunoglobulin isotype, (C) Durie-Salmon stage or (D) International Staging System (ISS) stage. Box plots show the 25th, 50th (median) and 75th percentiles with whiskers representing the 10th and 90th percentiles. Outliers are shown as circles. BJP, Bence Jones protein.

The serum level of prohepcidin did not differ significantly between patients at the time of diagnosis of MM and normal controls (121.3±32.4 vs. 130.8±17.8 ng/ml, P= 0.11; Fig. 1A). However, patients with mild or no renal insufficiency (CCr ≥50 ml/min) had significantly lower prohepcidin levels (116.1±31.2 ng/ml) when compared to healthy subjects (P=0.03). In addition, serum prohepcidin levels were not associated with the immunoglobulin isotype, Durie-Salmon stage or ISS stage (Fig. 1B, C and D).

### Correlation between serum prohepcidin levels and laboratory parameters

**Figure 2:**
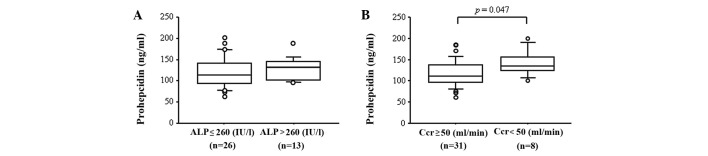
Serum prohepcidin levels in patients with multiple myeloma stratified by alkaline phosphatase (ALP) or creatinine clearance (CCr). (A) Serum prohepcidin levels in the elevated ALP group were similar to those in the normal ALP group. (B) Serum prohepcidin levels in the group with CCr <50 ml/min were significantly higher than those in the group with CCr ≥50 ml/min (P=0.047). Box plots show the 25th, 50th (median) and 75th percentiles with whiskers representing the 10th and 90th percentiles. Outliers are shown as circles.

The serum prohepcidin levels were weakly correlated with alkaline phosphatase (ALP; r=0.32, P=0.048; Table II). However, serum prohepcidin levels in patients in the abnormal ALP (>260 IU/l) group were similar when compared to patients in the normal ALP (≤260 IU/l) group (P=0.26, Fig. 2A). By contrast, although there was no correlation between prohepcidin levels and parameters of renal function, including blood urea nitrogen, creatinine, CCr or eGFR (Table II), patients with severe renal insufficiency (CCr <50 ml/min) had significantly higher prohepcidin levels when compared to patients with mild or no renal insufficiency (CCr ≥50 ml/min, P=0.047, Fig. 2B). These results indicate that serum prohepcidin levels were associated with ALP and renal function, but that these associations were weak. In addition, there was no correlation between prohepcidin levels and the percentage of bone marrow plasma cells, red blood cell count, hemoglobin, hematocrit, platelet count, serum levels of iron, ferritin, asparate aminotransferase (AST), alanine aminotransferase (ALT), LDH, uric acid, C-reactive protein, calcium, albumin and sβ2M (Table II). No correlation between levels of prohepcidin and serum cytokines, including IL-1β, IL-6 and TNF-α, was observed (Table II).

### Relevance of serum prohepcidin levels to prognosis

**Figure 3:**
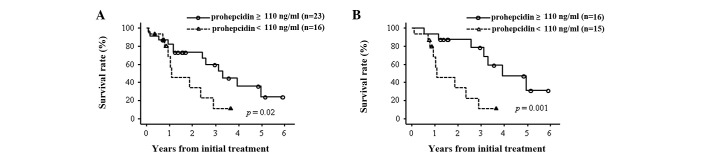
Kaplan-Meier estimates of overall survival (OS) based on serum prohepcidin levels. (A) The OS was significantly poorer in multiple myeloma patients with low serum prohepcidin (<110 ng/ml) as compared with those with high serum prohepcidin (≥110 ng/ml), regardless of renal function (P=0.02, log-rank test). (B) In MM patients with creatinine clearance ≥50 ml/min, the OS was significantly poorer in patients with low serum prohepcidin (<110 ng/ml) as compared to those with high serum prohepcidin (≥110 ng/ml; P=0.001, log-rank test).

The median follow-up among MM patients was 14.9 months (range, 0.9–71.5) and 19 of 39 patient mortalities occurred due to disease progression. To study the correlation between the prohepcidin level at diagnosis and the duration of survival, we divided the 39 MM patients into two groups according to prohepcidin levels and compared the survival rates. We designated patients as high-prohepcidin when the prohepcidin level was higher than 110 ng/ml, corresponding to one SD below the mean in normal controls, and low-prohepcidin when it was lower than 110 ng/ml. The overall survival of the 16 low-prohepcidin patients was significantly shorter than that of the 23 high-prohepcidin patients (median, 26.9 vs. 51.1 months, P=0.02; Fig. 3A).

We subsequently investigated whether prohepcidin was an independent prognostic factor in MM. A Cox proportional hazards model using established prognostic factors (24), along with levels of prohepcidin and several cytokines, including IL-1β, IL-6 and TNF-α, was generated. In the univariate analysis, platelet count, and serum levels of creatinine,serum calcium, sβ2M, performance status and prohepcidin were significant prognostic factors (Table III). In addition, a multivariate analysis showed that serum creatinine (HR, 5.32; 95% CI, 1.10–25.64), serum calcium (HR, 3.53; 95% CI, 1.01–12.33), performance status (HR, 4.15; 95% CI, 1.32–13.09) and serum prohepcidin (HR, 5.29; 95% CI, 1.65–17.03) were significant prognostic factors (Table III). We investigated whether prohepcidin was an independent prognostic factor for MM among the 31 patients with CCr ≥50 ml/min. The overall survival of the 15 low-prohepcidin patients with CCr ≥50 ml/min was significantly poorer than that of the 16 high-prohepcidin patients with CCr ≥50 ml/min (median, 26.9 vs. 92.3 months, P=0.001; Fig. 3B). Univariate analysis showed that platelet count, serum calcium, sβ2M and prohepcidin were significant prognostic factors. A multivariate analysis also showed that serum prohepcidin (HR, 5.65; 95% CI, 1.60–19.95) was a significant prognostic factor (Table IV).

## Discussion

Hepcidin regulates intestinal iron absorption and the release of iron from hepatic stores and from macrophages involved in the recycling of iron from hemoglobin (5). Hepcidin may also play a role in the anemia of patients with MM (19). However, the clinical significance of prohepcidin, the pro-hormone form of hepcidin, has not been elucidated in patients with MM. In this study, serum prohepcidin levels in MM patients were neither correlated with serum iron nor ferritin levels, but appeared to be associated with ALP levels and renal insufficiency (Table II and Fig. 2B). In contrast, low serum prohepcidin levels were independently associated with a poorer prognosis as compared to high serum prohepcidin levels in MM patients regardless of renal insufficiency. Although the sample size was insufficient, our study is the first to elucidate the clinical significance of serum prohepcidin levels in patients with MM.

Serum prohepcidin levels are not correlated with iron absorption in healthy individuals (25,26). Young *et al* reported that there was no significant correlation between serum hepcidin and serum prohepcidin levels in healthy women and that prohepcidin was not correlated with iron status (27). In addition, IL-6 was markedly increased within 3 h after injection of lipopolysaccharide (LPS), which is considered to be an upstream activator of inflammation in healthy individuals, and urinary hepcidin peaked within 6 h, followed by a significant decrease in serum iron (14). However, there were no significant changes in serum prohepcidin levels within a 22-h time frame in that study of healthy individuals (14). In MM patients with anemia, urinary, or serum hepcidin was positively correlated with serum ferritin and negatively with hemoglobin (19,28,29). By contrast, serum prohepcidin was not correlated with serum iron and ferritin in our subjects. These findings suggest that, similar to in healthy individuals, serum prohepcidin levels are not associated with serum hepcidin levels in MM patients, although urinary or serum hepcidin levels were not determined in the present study.

Hepcidin is specifically synthesized in the liver as an 84 amino acid prepropeptide and processed to its mature form in hepatocytes (30). It was reported that posttranslational processing of hepcidin in hepatocytes is mediated by the prohormone convertase furin, and the inhibition of furin activity prevents the conversion of prohepcidin to hepcidin, but does not inhibit its release from the cell (6). Serum prohepcidin levels in patients with chronic hepatitis C were positively correlated with serum ferritin (21) and IL-6 (20) levels. These results may indicate a positive correlation between serum prohepcidin and serum hepcidin levels in patients with chronic hepatitis C. By contrast, there was a negative correlation between serum prohepcidin and serum hepcidin levels in patients with inflammatory bowel disease (IBD) by univariate analysis (31). These conflicting results may indicate that the role of prohepcidin may differ in various diseases, including chronic hepatitis C, IBD and MM. The serum levels of prohepcidin and hepcidin, the expression of converting enzyme and iron status in each disease, including MM, should be further analyzed.

In the present study, serum prohepcidin levels were weakly associated with renal function as assessed by CCr, with significantly higher prohepcidin levels observed in patients with severe renal insufficiency (CCr <50 ml/min) when compared to patients with mild or no renal insufficiency (P= 0.047; Fig. 2B). Kulaksiz *et al* reported that in chronic renal insufficiency prohepcidin levels were significantly increased when compared with those in healthy controls, suggesting that the kidney may be involved in the metabolism or elimination of prohepcidin (13). Taes *et al* also reported that increased serum prohepcidin concentrations were observed with declining kidney function (32). These results suggest that renal function is one influencing factor on serum prohepcidin levels in the patients with MM in the present study, although this effect may be small in our study population.

The overall survival of the low-prohepcidin group was poorer than that of the high-prohepcidin group (Fig. 3A, Table III). Renal insufficiency is a known prognostic factor in MM (24) and the overall survival of the group with severe renal insufficiency (creatinine ≥2 mg/dl) in our study was also poorer (Table III). Prohepcidin levels may be inversely correlated with renal function (Table II) and the prohepcidin levels in the group of MM patients with severe renal insufficiency were significantly higher than those in the groups with mild or no renal insufficiency (Fig. 2B). In addition, seven out of eight patients with severe renal insufficiency (CCr <50 ml/min) were included in the high-prohepcidin group. When patients with severe renal insufficiency were excluded, the difference in overall survival between the high-prohepcidin group and low-prohepcidin group became more pronounced (Fig. 3B, P=0.001). Thus, although renal function should be considered in order to analyze the association between prohepcidin levels and overall survival, our results indicate that the poor prognosis of the low-prohepcidin patients group does not depend on renal insufficiency. The mechanism leading to a poor prognosis in patients with normal renal function and lower prohepcidin levels should be further examined.

In the present study, levels of prohepcidin and ALP, a known prognostic factor in MM (33), were correlated (Table II). This result is not consistent with lower prohepcidin levels being a poor prognostic factor in MM. In addition, we could not demonstrate any correlation between serum levels of prohepcidin and IL-6, TNF-α or IL-1β. Serum IL-6 is elevated in MM (18) and hepcidin mRNA was reportedly upregulated by IL-6 (16,17). The hepatic IL-6/STAT3 signal has a role in anemia of inflammation *in vivo* (34). IL-6 levels have also been reported as a prognostic factor in MM (18). However, levels of prohepcidin were not associated with IL-6, and IL-6 was not associated with prognosis in the present study. We suggest that expression of prohepcidin may not be regulated by these cytokines in MM and it is possible that serum levels of prohepcidin are more useful to prognosis than those of inflammatory cytokines or ALP in MM.

In conclusion, iron homeostasis may not affect serum prohepcidin levels in MM patients, but serum prohepcidin levels were weakly correlated with renal function and ALP. In addition, low serum prohepcidin may be an indicator for poor prognosis in MM patients regardless of their degree of renal function.

## Figures and Tables

**Table I:** Characteristics of the patients with multiple myeloma and healthy controls.

Variable	Multiple myeloma	Control
n	39	18
Age (years), mean ± SD	66.7±10.9	51.9±10.4
Age range (years)	43–85	30–67
Gender (male/female)	22/17	14/4
Immunoglobulin isotype		
IgG	23	
IgA	11	
IgD	1	
BJP	4	
Clinical stage		
Durie-Salmon		
II	7	
III	32	
ISS		
I	15	
II	11	
III	13	

BJP, Bence-Jones protein; ISS, International Staging System.

**Table II:** Correlation between serum prohepcidin and laboratory parameters.

Variable	Correlation coefficient	P-value
Red blood cell count	0.22	0.19
Hemoglobin	0.07	0.67
Hematocrit	0.10	0.53
Platelet count	0.15	0.36
Serum iron	−0.31	0.06
Ferritin	0.04	0.82
AST	−0.07	0.68
ALT	−0.10	0.55
ALP	0.32	0.048
Blood urea nitrogen	0.24	0.15
Creatinine	0.20	0.22
Uric acid	0.15	0.36
CCr	−0.23	0.16
eGFR	−0.25	0.13
CRP	0.11	0.49
Bone marrow plasma cells (%)	−0.02	0.91
Calcium	−0.07	0.67
Albumin	0.18	0.28
Sβ2M	0.02	0.92
IL-6	0.15	0.37
TNF-α	0.07	0.70
IL-1β	−0.02	0.91

AST, asparate aminotransferase; ALT, alanine aminotransferase; ALP, alkaline phosphatase; CCr, creatinine clearance; eGFR, estimate glomerular filtration rate; CRP, C-reactive protein; Sβ2M, serum β2-microglobulin; IL, interleukin; TNF, tumor necrosis factor.

**Table III:** Survival analysis in all multiple myeloma patients by Cox proportional hazards model.

		Univariate			Multivariate		
	n	HR	95% CI	P-value	HR	95% CI	P-value
Age (years)							
<65	16						
≥65	23	1.43	0.56–3.69	0.46			
Hemoglobin (g/dl)							
≥10	18						
<10	21	2.32	0.90–6.02	0.08			
Platelet (/*μ*l)							
≥130,000	30						
<130,000	9	4.02	1.37–11.82	0.01			
LDH							
normal	31						
abnormal	8	2.43	0.64–9.21	0.19			
ALP (IU/l)							
≤260	26						
>260	13	0.84	0.30–2.36	0.74			
Creatinine (mg/dl)							
<2	34						
≥2	5	4.29	1.14–16.18	0.03	5.32	1.10–25.64	0.02
Calcium (mg/dl)							
<10	32						
≥10	7	3.33	1.10–10.10	0.03	3.53	1.01–12.33	0.048
Albumin (g/dl)							
≥3.5	32						
<3.5	7	1.90	0.65–5.49	0.24			
Bone marrow plasma cells (%)							
<33	17						
≥33	22	2.39	0.89–6.41	0.08			
Sβ2M (mg/l)							
<3.5	18						
≥3.5	21	3.27	1.19–8.93	0.02			
Performance status							
1–3	34						
4	5	5.66	1.83–17.50	<0.001	4.15	1.32–13.09	0.02
Prohepcidin (ng/ml)							
≥110	23						
<110	16	3.18	1.18–8.55	0.02	5.29	1.65–17.03	0.01
IL-6 (pg/ml)							
<4	18						
≥4	19	1.58	0.61–4.09	0.35			
TNF-α (pg/ml)							
<15.6	25						
≥15.6	11	0.86	0.32–2.31	0.77			
IL-1β (pg/ml)							
<10	31						
≥10	1	7.07	0.79–63.29	0.08			

HR, hazard ratio; CI, confidence interval; LDH, lactate dehydrogenase; ALP, alkaline phosphatase; Sβ2M, serum β2-microglobulin; IL, interleukin; TNF, tumor necrosis factor.

**Table IV:** Survival analysis in multilpe myeloma patients without severe renal insufficiency by Cox proportional hazards model.

		Univariate			Multivariate		
	n	HR	95% CI	P-value	HR	95% CI	P-value
Age (years)							
<65	14						
≥65	17	1.76	0.60–5.16	0.31			
Hemoglobin (g/dl)							
≥10	18						
<10	13	2.01	0.72–5.60	0.18			
Platelet count (/*μ*l)							
≥130,000	26						
<130,000	5	5.33	1.38–20.55	0.02			
LDH							
normal	27						
abnormal	4	4.23	0.83–21.53	0.08			
ALP (IU/l)							
≤260	21						
>260	10	0.97	0.30–3.09	0.95			
Calcium (mg/dl)							
<10	28						
≥10	3	6.61	1.19–36.81	0.03	3.63	0.65–20.26	0.14
Albumin (g/dl)							
≥3.5	27						
<3.5	4	1.87	0.50–6.94	0.35			
Bone marrow plasma cells (%)							
<33	15						
≥33	16	2.29	0.76–6.86	0.14			
Sβ2M (mg/l)							
<3.5	18						
≥3.5	13	2.98	1.02–8.74	0.046			
Performance status							
1–3	29						
4	2	4.49	0.93–21.72	0.06			
Prohepcidin (ng/ml)							
≥110	16						
<110	15	6.24	1.82–21.35	0.004	5.65	1.60–19.95	0.01
IL-6 (pg/ml)							
<4	15						
≥4	14	1.29	0.44–3.76	0.64			
TNF-α (pg/ml)							
<15.6	18						
≥15.6	10	0.78	0.26–2.30	0.65			
IL-1β (pg/ml)							
<10	23						
≥10	1	10.99	1.00–121.24	0.05			

HR, hazard ratio; CI, confidence interval; LDH, lactate dehydrogenase; ALP, alkaline phosphatase; Sβ2M, serum β2-microglobulin; IL, interleukin; TNF, tumor necrosis factor.
